# Neutralizing epitopes on *Clostridioides difficile* toxin A revealed by the structures of two camelid VHH antibodies

**DOI:** 10.3389/fimmu.2022.978858

**Published:** 2022-11-16

**Authors:** Baohua Chen, Kay Perry, Rongsheng Jin

**Affiliations:** ^1^ Department of Physiology and Biophysics, School of Medicine, University of California, Irvine, CA, United States; ^2^ NE-CAT, Advanced Photon Source, Argonne National Laboratory, Argonne, IL, United States; ^3^ Department of Chemistry and Chemical Biology, Cornell University, Argonne, IL, United States

**Keywords:** *Clostridioides difficile*, *C. difficile* infection, TcdA, TcdB, large clostridial glucosylating toxin, VHH, antibody, antitoxin

## Abstract

Toxin A (TcdA) and toxin B (TcdB) are two key virulence factors secreted by *Clostridioides difficile*, which is listed as an urgent threat by the CDC. These two large homologous exotoxins are mainly responsible for diseases associated with *C. difficile* infection (CDI) with symptoms ranging from diarrhea to life threatening pseudomembranous colitis. Single-domain camelid antibodies (VHHs) AH3 and AA6 are two potent antitoxins against TcdA, which when combined with two TcdB-targeting VHHs showed effective protection against both primary and recurrent CDI in animal models. Here, we report the co-crystal structures of AH3 and AA6 when they form complexes with the glucosyltransferase domain (GTD) and a fragment of the delivery and receptor-binding domain (DRBD) of TcdA, respectively. Based on these structures, we find that AH3 binding enhances the overall stability of the GTD and interferes with its unfolding at acidic pH, and AA6 may inhibit the pH-dependent conformational changes in the DRBD that is necessary for pore formation of TcdA. These studies reveal two functionally critical epitopes on TcdA and shed new insights into neutralizing mechanisms and potential development of epitope-focused vaccines against TcdA.

## Introduction

Infections caused by the Gram-positive, spore-forming bacterium *Clostridioides difficile* (*C. difficile*) is one of the most common health care-associated infections (1, 2). According to a recent CDC report, there were ~12,800 deaths and ~$1B healthcare costs attributable to *C. difficile* infection (CDI) (3). Antibiotics including metronidazole, vancomycin, and fidaxomicin are currently the primary treatment options for CDI. However, up to 30% of patients suffer a recurrence following initial antibiotics therapy and the risks of additional recurrences also increase (4), which raises the urgent need to develop more effective therapeutics for CDI treatments.

**Figure 1:**
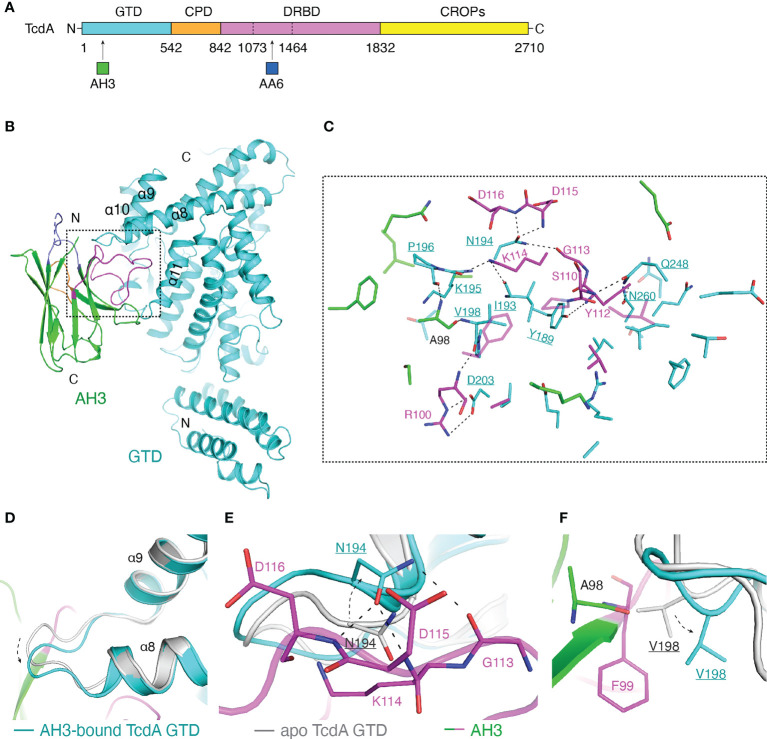
Structure of the TcdA GTD-AH3 complex. **(A)** Schematic diagram of TcdA showing its domain organization and the VHH-binding sites. TcdA: GTD (cyan), CPD (orange), DRBD (violet), and CROPs (yellow); AH3 (green); AA6 (marine). **(B)** A cartoon representation of the TcdA GTD(cyan)-AH3(green) complex. The CDR1, CDR2, and CDR3 of AH3 are colored slate, orange, and magenta, respectively. **(C)** A close-up view of the TcdA GTD-AH3 complex interface. The interface residues are colored as in panel **B**, while the GTD residues are underlined. **(D)** AH3 binding induces a ~2.1 Å shift to a loop connecting the α8 and α9 helices of TcdA GTD (cyan) when compared to that of the standalone GTD (gray, PDB code: 3SRZ). AH3 is colored as in panel **(B)**. **(E)** Residue N194 of the AH3-bound TcdA GTD shifts ~2.9 Å to avoid clashing with K114 of AH3 and also establish three pairs of hydrogen bonds with G113, D115, and D116 of AH3. **(F)** Residue V198 of the AH3-bound TcdA GTD shifts ~2.6 Å to avoid clashing with A98 and F99 of AH3.

Two high-molecular-weight exotoxins, toxin A (TcdA) and toxin B (TcdB), secreted by *C. difficile* are the major virulence factors responsible for CDI, which lead to a wide spectrum of clinical symptoms ranging from mild diarrhea to life threatening pseudomembranous colitis (4–7). The sequences and three dimensional structures of TcdA and TcdB show a similar modular arrangement which could be divided into four functional domains: an amino-terminal glucosyltransferase domain (GTD), a cysteine protease domain (CPD), a delivery and receptor-binding domain (DRBD), and a carboxy-terminal combined repetitive oligopeptides domain (CROPs) (8–10) (**Figure 1A**). TcdA and TcdB are internalized into host cells *via* receptor-mediated endocytosis (5, 10–14). Acidification in the endosomes then triggers a hydrophobic pore-forming region in the DRBD to undergo conformational rearrangements (15–18) in order to deliver the GTD and the CPD across endosome membrane into the cytosol, where the CPD cleaves the GTD upon binding inositol hexakisphosphate (InsP6) (19, 20). The liberated GTD targets and inactivates Rho and/or Ras families of small guanosine triphosphatases (GTPases) in the host cells by glucosylating the key threonine residue in the GTPases and inhibiting their functions as molecular switches, which leads to disruption of the actin cytoskeleton, cell rounding, and eventually cell death (21–25).

The complex multistep process of intoxication in fact provides us many opportunities for therapeutic interventions for CDI. For example, many neutralizing antibodies have been developed, which target TcdA or TcdB and inhibit their cellular toxicity by blocking the functions of individual toxin fragments during the intoxication cascade (26–30). These efforts have led to the successful commercialization of an anti-TcdB monoclonal antibody (mAb), bezlotoxumab, which neutralizes TcdB by targeting the TcdB CROPs and blocking the binding of a crucial host receptor CSPG4 (11, 27, 31). Several neutralizing mAbs against TcdA, such as actoxumab and PA50, have also been reported that target the CROPs and block TcdA from binding to cell surface receptors (27, 32, 33).

In addition to the conventional mAbs, great efforts have been invested to develop camelid heavy chain only antibodies (VHHs) as antitoxins against TcdA and TcdB because of their small sizes, high affinity, specificity, and stability (34, 35). For example, in earlier studies we found that VHH 5D binds to the pore-forming region in TcdB DRBD and prevents the pH-induced pore formation of the toxin, and VHH E3 binds to the N-terminal four-helix bundle in TcdB GTD that may interfere with membrane association of the GTD (10). Several TcdA-targeting neutralizing VHHs have also been developed, including A20.1 and A26.8 that bind to the CROPs, AH3 that binds to the GTD, and AA6 that binds to the CPD-DRBD (26, 36). AH3 and AA6 are particularly interesting because they neutralized the cytopathic effects of TcdA at nanomolar concentrations (26). Furthermore, when AH3 and AA6 were combined with two TcdB-neutralizing VHHs (E3 and 5D) in a form of single-chain hetero-tetrameric VHH design, they showed potent protection against primary and recurrent CDI in animal models (26, 28, 30). Here we reported the co-crystal structures of VHH AH3 and AA6 in complex with TcdA GTD (residues 1-542) and a fragment of TcdA DRBD (residues 1073-1464, termed as TcdA^1073-1464^), respectively. These structures reveal two distinct mechanisms by which antibodies neutralize TcdA by inhibiting the unfolding of the GTD or the pore formation in the DRBD, two crucial steps during the intoxication cascade.

## Materials and methods

### Protein expression and purification

The genes encoding the two VHHs (AH3, AA6), TcdA GTD (residues 1-542, strain VPI10463) and a truncated DRBD of TcdA (residues 1073-1464, termed as TcdA^1073-1464^, strain VPI10463) were cloned into a modified pET28a vector that has a 6×His/SUMO tag introduced to the N-terminus *via BamH* I/*Xho* I restriction sites. TcdA GTD carries a K190A mutation to minimize degradation during protein expression and purification as previously described (22).

The recombinant proteins were overexpressed in *E. coli* strain BL21-star (Invitrogen). Bacteria were cultured at 37°C in LB medium containing kanamycin. Protein expression was induced with 1 mM isopropyl-β-D-thiogalactopyranoside (IPTG) when cell density (OD_600_) reached ~0.8. The temperature was then reduced to 18°C, and the protein expression continued at 18°C for 18 hours. Cells were harvested by centrifugation and stored at -80°C for future use.

For purification, cell pellets were re-suspended in a buffer containing 50 mM Tris, pH 8.5, 400 mM NaCl, 30 mM imidazole and lysed by sonication. The His-tagged proteins were purified by Ni-NTA affinity resins (Qiagen), and the bound proteins were eluted with a similar buffer containing 400 mM imidazole, pH 8.5. The His/SUMO tag was cleaved by SUMO-protease. Proteins were then exchanged to a buffer containing 20 mM Tris, 40 mM NaCl, pH 8.5, and subjected to Mono-Q ion-exchange chromatography (GE Healthcare) using a NaCl gradient. The peak fractions were pooled, concentrated to ~10 mg/ml and stored at -80°C until future use.

The TcdA GTD-AH3 and the TcdA^1073-1464^-AA6 complexes were assembled by mixing the purified TcdA GTD and TcdA^1073-1464^ with AH3 and AA6, respectively, at a molar ratio of 1:1.5 for 3 hours on ice. The complexes were further purified using a Mono-Q ion-exchange chromatography (GE Healthcare) using a NaCl gradient. For crystallization, the TcdA GTD-AH3 complex was exchanged to a buffer containing 20 mM Tris, 150 mM NaCl, pH 8.0, 2 mM UDP-glucose, 2 mM MnCl_2_, and the TcdA^1073-1464^-AA6 complex was exchanged to a buffer containing 20 mM Tris, 150 mM NaCl, pH 8.0. The two complexes were concentrated to ~10 mg/ml and stored at -80°C until future use.

### Crystallization

Initial crystallization screening of the TcdA GTD-AH3 and the TcdA^1073-1464^-AA6 complexes were carried out at 18°C using a Gryphon crystallization robot (Art Robbins Instruments) with high-throughput sparse matrix screening kits (Hampton Research and Qiagen) using the sitting-drop vapor diffusion method (0.3 µl protein + 0.3 µl reservoir equilibrated against 55 µl reservoir). Crystal optimizations were carried out using the hanging-drop vapor diffusion method at 18°C by mixing equal volume of protein and reservoir solutions. The best crystals of the TcdA GTD-AH3 complex were obtained in a condition containing 0.2 M potassium phosphate and 20% (w/v) PEG 3350. The best crystals of the TcdA^1073-1464^-AA6 complex were obtained in a condition with 0.2 M ammonium acetate, 0.1 M Bis-Tris, pH 5.7, and 15% PEG 3350. Crystals were cryoprotected in the mother liquor supplemented with 15% (v/v) glycerol and snap frozen in liquid nitrogen for data collection.

### Data collection and structure determination

The X-ray diffraction data were collected at 100 K at the NE-CAT beamline 24-ID-E, Advanced Photon Source. The data were processed using XDS as implemented in RAPD (https://github.com/RAPD/RAPD) (37). The structure of the TcdA GTD-AH3 complex was solved by molecular replacement using the structures of TcdA GTD (PDB 7U2P) (22) and VHH 5D (PDB 6OQ5) (10) as search models *via* PHENIX.Phaser-MR (38). The structure of the TcdA^1073-1464^-AA6 complex was solved by molecular replacement using the structures of the corresponding fragment (1073–1464) in TcdA DRBD (PDB code: 7U1Z) (8) and a VHH (PDB 5M2J) (39) as search models *via* PHENIX.Phaser-MR (38). All refinement and model building procedures were carried out with PHENIX.refine (40) and COOT (41). All the refinement progress was monitored with the free R value using a 5% randomly selected test set (42). The structure was validated by MolProbity (43). **Table S1** shows the detailed statistics of data collection and refinement. All the structure figures were prepared by PyMOL (DeLano Scientific). Calculations of the buried molecular surface area and the shape complementarity were carried out using PISA (Proteins, Interfaces, Structures and Assemblies) program (44) and SC in CCP4 program (45, 46), respectively.

### Protein melting assay

The thermal stability of TcdA GTD and the TcdA GTD-VHH complexes was measured using a fluorescence-based thermal shift assay on a StepOne real-time PCR machine (Life Technologies). TcdA GTD with or without VHHs (molar ratio of 1:1.2) were incubated on ice in a buffer containing 150 mM NaCl and either 50 mM sodium acetate (pH 5.0-5.5) or 50 mM Bis-Tris (pH 6.0-6.5) for 2 hours. Immediately before the experiment, proteins (~0.25 mg/ml) were mixed with the fluorescent dye SYPRO Orange (Sigma-Aldrich), and the mixtures were heated from 25°C to 95°C in a linear ramp. The midpoint of the protein-melting curve (T_m_) was determined using the analysis software provided by the instrument manufacturer. Data obtained from three independent experiments were averaged to generate the graph.

### ANS (8-anilinonaphthalene-1-sulfonic acid) binding assay

One µM of TcdA GTD, TcdA GTD-AH3, TcdA GTD-AA6 (molar ratio of 1:1), or AH3 was incubated with 200 µM of ANS for 20 min in either 50 mM sodium acetate (pH 4.0-4.6) or 50 mM sodium citrate (pH 5.0-6.5). One and half µM of TcdA^1073-1464^, TcdA^1073-1464^-AA6, TcdA^1073-1464^-AH3 (molar ratio of 1:1), or AA6 was incubated with 375 µM ANS for 15 min in either 50 mM sodium acetate (pH 4.0-4.6) or 50 mM sodium citrate (pH 5.0-6.5). All buffers contained 150 mM NaCl. Fluorescence intensity was recorded at 25°C using a Molecular Devices SpectraMax M2e spectrophotometer with excitation at 366 nm and emission at 480 nm. The fluorescence intensity was corrected by subtraction of background fluorescence from ANS in a buffer without protein. Error bars indicate SD of three replicate measurements.

## Results

### Crystal structure of VHH AH3 in complex with the GTD of TcdA

A previous study showed that AH3 binds to the GTD of TcdA (26). We successfully crystallized a complex composed of the GTD of TcdA (residues 1-542) and AH3 in the presence of UDP-glucose and Mn^2+^. The crystals belong to the space group P12_1_1 with cell dimensions of a = 80.12 Å, b = 131.64 Å, c = 83.80 Å, α = 90°, β = 110.02°, γ = 90°. The structure of the TcdA GTD-AH3 complex was determined to 2.10-Å resolution by molecular replacement using TcdA GTD (PDB code: 7U2P) and VHH 5D (PDB code: 6OQ5) as the searching models (**Figure 1B**) (see Materials and Methods). The secondary structures focusing on the interacting regions of the TcdA GTD-AH3 complex are shown in **Figure S1**. Statistics of data collection and refinement are shown in **Table S1**.

The overall architecture of the AH3-bound TcdA GTD is similar to that of the UDP-glucose-bound TcdA GTD (PDB code: 3SRZ, referred to as apo TcdA GTD) (47), with a root mean square deviation (RMSD) of ~0.522 Å over 387 residues. Most of the interactions are mediated by the complementarity-determining region 3 (CDR3) of AH3 that inserts into a groove formed between the α8 and α11 helices of TcdA GTD (**Figure 1B**), burying an interface area of ~909.6 Å^2^ with a shape complementarity score of 0.760 (45). The core of the TcdA GTD-AH3 interface is mainly mediated by extensive hydrogen bond interactions, complemented with salt bridge and van del Waals interactions (**Figure 1C** and **Table S2**). Of note, a loop linking the α8 and α9 helices of TcdA GTD shifts ~2.1 Å compared to the apo state in order to better accommodate the binding of AH3 (**Figure 1D**). As a result, N194 of TcdA GTD shifts ~2.9 Å to avoid a potential clash with K114 of AH3 and also establish three pairs of hydrogen bonds with the main chains of G113, D115 and D116 of AH3 (**Figure 1E**), while V198 of TcdA GTD takes a ~2.6 Å movement to avoid potential clashes with A98 and F99 of AH3 (**Figure 1F**). A few other TcdA amino acids in this area such as Y189, I193, K195, P196, and D203 also form hydrogen bond and salt bridge interactions with the CDR3 of AH3 (**Figure 1C**). Complementing this core interface, twelve TcdA amino acids around the α11 helix and the connecting loop between α10 and α11 mostly contribute hydrophobic interactions with the CDR3 of AH3 to further stabilize the complex (**Table S2**).

### Crystal structure of VHH AA6 in complex with a fragment of the DRBD

**Figure 2:**
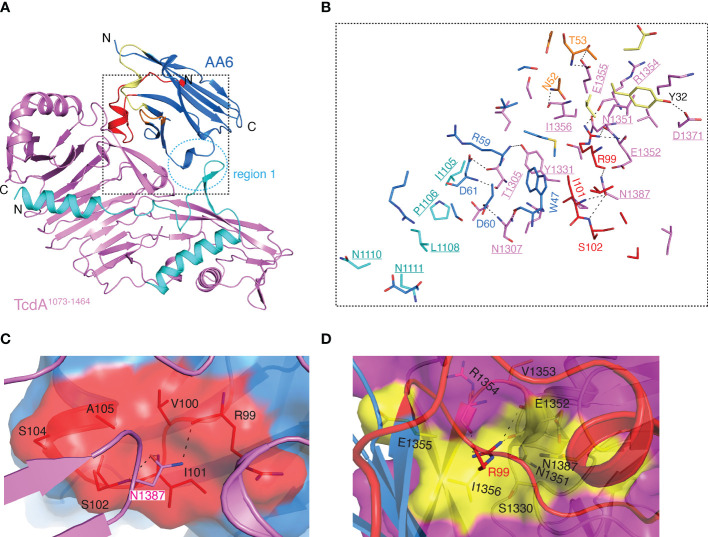
Structure of the TcdA^1073-1464^-AA6 complex. **(A)** A cartoon representation of the TcdA^1073-1464^-AA6 complex. TcdA^1073-1464^ and AA6 are colored violet and marine, respectively, while the pore-forming region of TcdA is colored cyan. The CDR1, CDR2, and CDR3 of AA6 are colored pale yellow, orange, and red, respectively. The region 1 of the interface is highlighted with a dashed circle. **(B)** A close-up view of the TcdA^1073-1464^-AA6 interface. The interacting residues are colored as in panel **A**, while the TcdA residues are underlined. **(C)** Residue N1387 of TcdA^1073-1464^ inserts into a pocket in AA6 that is formed by residues R99, V100, I101, S102, S104, and A105. AH3 is shown as a surface presentation and colored as in panel **(A)**. **(D)**. Residue R99 of AA6 inserts into a pocket in TcdA that is formed by residues S1330, N1351-I1356, and N1387. TcdA^1073-1464^ is shown as a surface presentation and colored as in panel **A**, while TcdA residues that interact with AA6-R99 are colored yellow.

The epitope of AA6 was mapped to be within the CPD-DRBD of TcdA in a prior study (26). We examined AA6 binding to a series of recombinant TcdA fragments using a pull-down assay, and we found that a fragment of TcdA DRBD (residues 1073-1464, termed as TcdA^1073-1464^) was sufficient for AA6 binding and most suitable for crystallization (**Table S3**). The TcdA^1073-1464^-AA6 complex was successfully crystallized in space group P12_1_1 with cell dimensions of a = 54.02 Å, b = 86.00 Å, c = 109.34 Å, α = 90°, β = 91.45°, γ = 90°. The structure of the TcdA^1073-1464^-AA6 complex was determined to 1.81-Å resolution by molecular replacement using the fragment structure of TcdA^1073-1464^ adapted from the structure of TcdA in PDB 7U1Z and a VHH in PDB 5M2J as the searching models (**Figure 2A**). A near complete structure of TcdA^1073-1464^ including residues 1080-1464 was built except for the first seven N-terminal residues that had no visible electron density. The secondary structures focusing on the interacting regions of the TcdA^1073-1464^-AA6 complex are shown in **Figure S2**. Statistics of data collection and refinement are shown in **Table S1**.

The overall structure of the AA6-bound TcdA^1073-1464^ is similar to that of the standalone TcdA fragment in the context of the holotoxin with a RMSD of ~0.576 Å over 313 residues, indicating that AA6 does not force appreciable conformational changes in the toxin (8). Residues from all three CDRs of AA6 contribute to direct interactions with TcdA^1073-1464^ with the CDR3 playing a dominating role (**Figure 2A**). Complementing the CDRs, some AA6 residues in the framework region FR3 are also engaged in TcdA binding. The complex buries a total interface area of ~957.8 Å^2^ with a shape complementarity score of 0.696 (45). The structure reveals that AA6 recognizes two regions in TcdA^1073-1464^ that are well separated on the primary sequence but converge on the 3D: residues I1105-N1111 (region 1) and T1305-N1387 (region 2) (**Figure 2A**). Of note, the region 1 is a part of the pore-forming region of TcdA, which includes residues 958 to 1130 in the holotoxin. The pore-forming region is structurally buried and therefore protected in the DRBD under neutral pH but released upon endosome acidification in order to facilitate the translocation of the GTD and the CPD to cytosol (10, 15–18, 48). AA6 binding stabilizes the conformation of this part of the pore-forming region *via* mostly van del Waals interactions involving AA6 residues D61, S62, R66, and E88 in the FR3 and TcdA residues I1105, P1106, L1108, N1110, and N1111 (**Figure 2B** and **Table S4**). The region 2 of TcdA mediates most of the AA6 binding *via* extensive interactions with all three CDRs. It is worth noting that N1387 of TcdA^1073-1464^ is likely a key epitope for AA6 as it points toward a pocket in AA6 that consists of residues R99, V100, I101, S102, S104 and A105 in the CDR3, forming three pairs of hydrogen bonds and two van del Waals interactions (**Figure 2C** and **Table S4**). Inversely, residue R99 in the CDR3 of AA6 binds to a pocket in TcdA that is composed of residues S1330, N1351-I1356, and N1387, establishing four pairs of hydrogen bonds and four van del Waals interactions (**Figure 2D** and **Table S4**).

### AH3 interferes with the pH-dependent unfolding of the GTD

**Figure 3:**
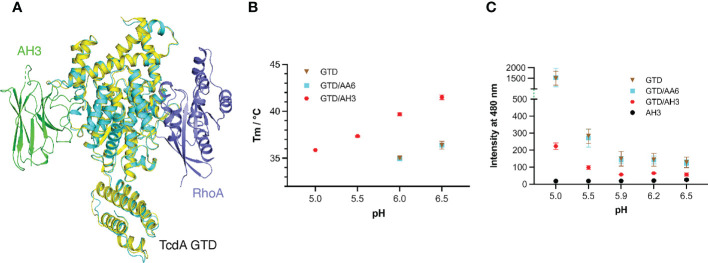
AH3 interferes with the pH-dependent unfolding of the GTD. **(A)** The structures of the TcdA GTD(cyan)-AH3(green) complex and the TcdA GTD(pale yellow)-RhoA (slate, PDB code: 7U2P) complex are superimposed based on the GTD, which reveals that AH3 does not directly affect substrate binding of the GTD. **(B)** Thermal stability of the standalone TcdA GTD (brown) and GTD in the presence of AA6 (cyan) or AH3 (red). The data are presented as mean ± SD; n = 3. **(C)** pH-dependent unfolding of TcdA GTD probed by ANS. The mean values of fluorescence intensity at 480 nm are shown. The data are presented as mean ± SD; n = 3.

Previous studies focused on the neutralizing epitopes on the GTD of TcdB have revealed several distinct neutralizing mechanisms (49). For example, VHH 7F and E3 inhibit the autoprocessing and disrupt the plasma membrane association of the GTD of TcdB, respectively (10, 50). A humanized mAb PA41 neutralizes TcdB by preventing the delivery of its GTD into the cytosol of host cells (51). In contrast, to the best of our knowledge, AH3 is the only known neutralizing antibody that targets TcdA GTD (26), and its neutralizing mechanism remains to be defined. Our crystal structure shows that the epitope of AH3 is located on the opposite side of the substrate-binding site of the GTD, and therefore it should not directly affect the glucosylation of Rho GTPases (22) (**Figure 3A**). Another unique feature of the GTD is that it will partially unfold when triggered by acidification in the endosomes and subsequently translocate to the cytosol with the help from the pore-forming region in the DRBD (5). Researches focusing on other pore-forming toxins like botulinum toxin, diphtheria toxin, and anthrax toxin show that partial unfolding of their enzymatic domains is required for their translocation to the cytosol (52–56). Therefore, we hypothesized that AH3 binding may interfere with the pH-dependent conformational change and unfolding of the GTD.

To this end, we examined the effect of AH3 on the thermo-stability of the GTD using a fluorescence-based thermal shift assay with AA6 as a negative control. The melting temperature (T_m_) of TcdA GTD was 36.4°C and 34.9°C at pH 6.5 and 6.0, respectively, but no clear melting phase could be observed at pH 5.5 and 5.0, indicating that the GTD is partially unfolded at pH below 6 (**Figure 3B**). In the presence of AH3, the T_m_ of the GTD increased by 5.1°C and 4.8°C at pH 6.5 and 6.0, respectively, and the stability of the GTD at pH 5.5 and 5.0 increased drastically as evidenced by its T_m_ values at 37.3°C and 35.9°C, respectively. In an independent assay, we monitored the effect of AH3 on acidic pH-induced unfolding of TcdA GTD using 8-anilinonaphthalene-1-sulfonic acid (ANS), a fluorescent hydrophobic dye commonly used to probe protein folding (57). Large increases in fluorescence intensity were observed at pH below 5.5, which were caused by exposure of hydrophobic surfaces during TcdA GTD unfolding. However, the GTD unfolding was greatly inhibited by AH3 but not AA6 (**Figure 3C**). The structure of the GTD-AH3 complex reveals that the CDR3 of AH3 simultaneously interacts with residues located in the α8, α9, and α11 helices of TcdA GTD, as well as loops connecting α8-α9 and α10-α11, which could help enhance the stability of the GTD (**Figures 1B, C** and **Table S2**). These data suggest that AH3 stabilizes the structure of the GTD upon binding, which inhibits the pH-dependent unfolding of the GTD and therefore impedes the delivery of the GTD to the cytosol. Similar neutralizing mechanisms have been reported for VHHs targeting ricin and botulinum toxin, where VHHs binds to the protein cargos and inhibit their delivery (58–60).

### AA6 may block the pore formation of TcdA

**Figure 4:**
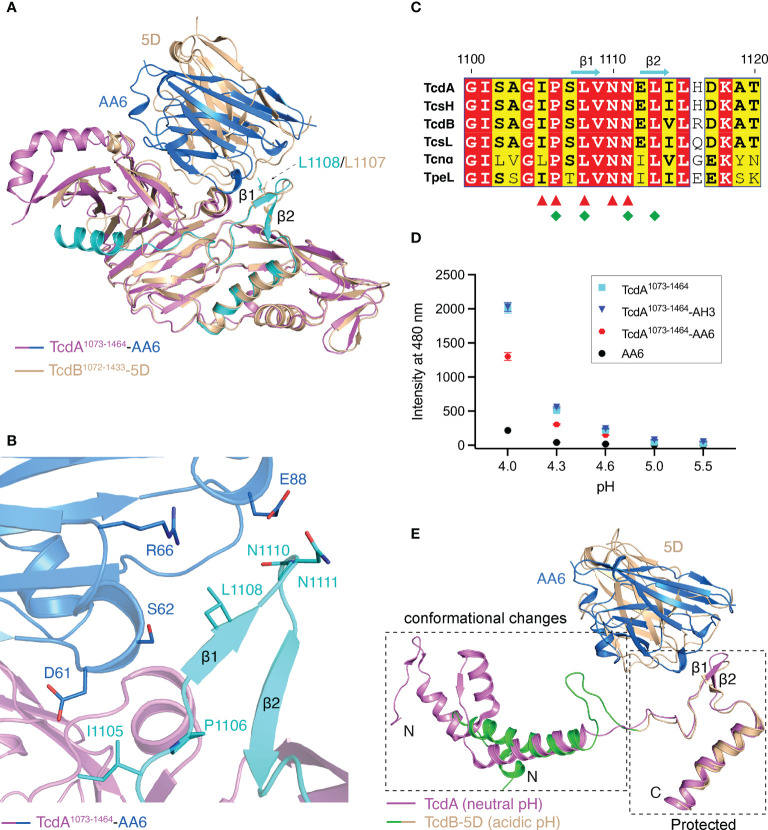
AA6 may block the pore formation of TcdA. **(A)** The structures of the TcdA^1073-1464^-AA6 complex and the TcdB^1072-1433^-5D complex (PDB code: 6OQ6) are superimposed based on TcdA and TcdB. The TcdA^1073-1464^-AA6 complex is colored violet and marine, respectively, while the TcdB^1072-1433^-5D complex is colored wheat. The binding site of AA6 in TcdA and that of 5D in TcdB are largely overlapping, while both grip the β hairpin motif in the pore-forming region. **(B)** AA6 (marine) interacts with the pore-forming region (cyan) of TcdA (violet). **(C)** Sequence alignment of the pore-forming region among different members in the LCGT family focusing on the AA6/5D-binding sites. The AA6-binding residues in TcdA and the 5D-binding residues in TcdB are indicated by red triangles and green rhombuses, respectively. **(D)** pH-dependent conformational change of TcdA^1073-1464^ probed by ANS. The mean values of fluorescence intensity at 480 nm are shown. The data are presented as mean ± SD; n = 3. **(E)** The pore-forming region of TcdA at neutral pH (pink, PDB code: 7U1Z) and that of TcdB at acidic pH (green and wheat, PDB code: 6OQ5) adopt markedly different conformations. The conformational changes are restricted in a region N-terminal to the 5D/AA6 binding site.

After determining the structure of the TcdA^1073-1464^-AA6 complex, we were surprised to notice that the AA6-binidng site in TcdA is largely overlapping with that of a potent neutralizing VHH, 5D, in TcdB^1072-1433^ (10) (**Figure 4A**). Furthermore, both VHHs bind to a key section of the pore-forming region (e.g., residues I1105-N1111 in TcdA), where they grip a β hairpin motif (β1-β2) (**Figures 4B, C**).The importance of this region to the cellular toxicity is evidenced by a prior study showing that mutating just one residue in this region of TcdB, L1107K (equivalent to L1108 in TcdA), caused a >1,000-fold reduce of toxicity (10, 15). Our prior studies on 5D and TcdB showed that 5D inhibits the acidic pH-induced conformational changes and pore formation mediated by the pore-forming region of TcdB (10). Intrigued by these findings, we examined how AA6 may affect the structure of TcdA^1073-1464^ at acidic pH using the hydrophobic dye ANS. We found that AA6 significantly inhibited the unfolding of TcdA^1073-1464^ at pH below 4.3, which is consistent with the effect of 5D on TcdB, suggesting that AA6 may block the pore formation of TcdA (**Figure 4D**).

It is widely accepted that the pore-forming region of TcdA/TcdB, which carries a large number of hydrophobic residues, is protected by the DRBD at neutral pH, but partially unfolds and detaches from the DRBD when induced by acidic endosomal pH in order to form a pore (5, 10, 48). Drastic conformational changes could be observed in the pore-forming regions when comparing the structure of TcdA obtained at the neutral pH and a structure of TcdB that was obtained under acidic pH and represents a pore-forming intermediate state (**Figure 4E**) (8–10). Nevertheless, it has been shown that 5D is able to grip and stabilize a β hairpin motif in TcdB’s pore-forming region, which may subsequently block the acidic pH-induced unfolding of TcdB (10). We found that AA6 grips the β hairpin motif in the pore-forming region of TcdA in a way that is highly similar to what 5D does to TcdB (**Figure 4E**), which suggests that AA6 may constrain the conformation of this β hairpin motif in TcdA and inhibit the conformational changes necessary for pore formation at endosomal pH. Further studies are warranted to verify this structural finding in the context of TcdA holotoxin. Notably, this part of the pore-forming region recognized by AA6 and 5D is highly conserved among a family of large clostridial glucosylating toxins (LCGTs), which include TcdA and TcdB, *C. novyi* α-toxin (Tcnα), *C. sordellii* lethal and hemorrhagic toxins (TcsL and TcsH), and *C. perfringens* toxin (TpeL) (5, 10, 15, 61) (**Figure 4C**), suggesting it could be a good target for the development of broad-spectrum vaccines and antibodies targeting LCGTs.

## Discussion

In this study we identified two novel neutralizing epitopes in TcdA that are the targets of VHH AH3 and AA6. Our structural and biochemical studies suggest that AH3 binds to the GTD and enhances its overall stability, and therefore impedes its unfolding at acidic pH, and that AA6 binds to the DRBD and blocks the conformational changes in the pore-forming region of TcdA. We found that the binding mode and neutralizing mechanism of AA6 on TcdA are remarkably similar to that of VHH 5D on TcdB (10). Since AA6 and 5D were raised in two alpacas that were immunized with the full length TcdA and TcdB, respectively, and panned independently (26), this finding suggests that this conserved epitope for AA6 and 5D is functionally critical for both TcdA and TcdB and therefore a good candidate for the development of vaccines and therapeutic antibodies.

**Figure 5:**
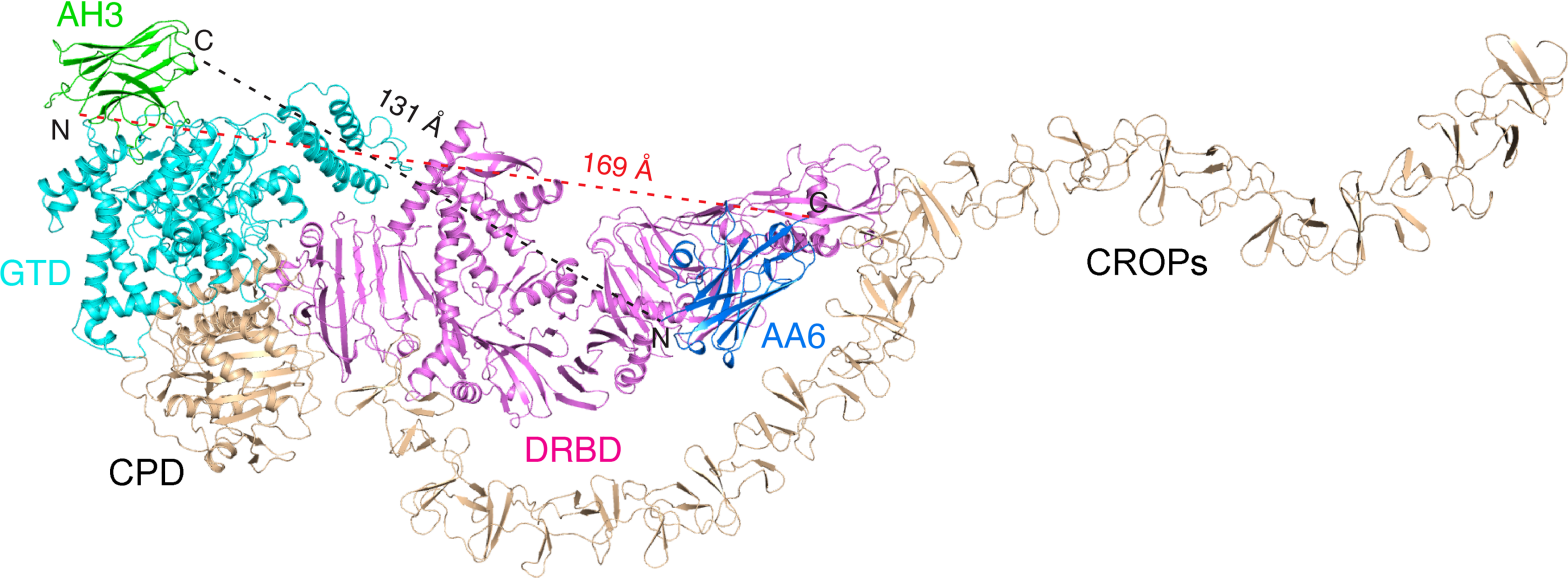
Mapping the AH3- and AA6-binding sites on TcdA holotoxin. AH3 (green) and AA6 (marine) bind to two distinct regions on TcdA holotoxin (GTD: cyan, DRDB: violet, other domains: wheat). The linear distance between the C-terminus of AH3 to the N-terminus of AA6 is ~131 Å and ~169 Å vice versa.

Membrane translocation and delivery of the GTD to the host cell cytosol is an obligatory step for TcdA intoxication. Therefore, antibodies blocking this step should provide potent protection. We found that AH3 and AA6 can bind simultaneously on TcdA holotoxin (8, 9) (**Figure 5**). Therefore, combining AH3 and AA6 in a hybrid molecule should lead to even higher neutralizing potency because of synergistic binding of both VHHs. In fact, several multivalent VHHs that combined AH3 and AA6 with two anti-TcdB VHH E3 and 5D into a single molecule have been developed, which dramatically outperformed the individual VHHs when neutralizing TcdA and TcdB (26, 28, 30). In these multivalent VHHs, the four VHHs were linked together *via* short peptide linkers in different orientations, such as AH3–E3–E3–AA6 (26), AH3–5D–E3–AA6 (28), and AH3–5D–AA6–E3 (the dash lines indicate peptide linkers) (30). In the context of TcdA holotoxin, the shortest linear distance between the C-terminus of AH3 to the N-terminus of AA6 is ~131 Å if AH3 is linked to the N-terminal of AA6, and ~169 Å vice versa (**Figure 5**). An ideal peptide linker should have a sufficient length and flexibility to allow simultaneous binding of AH3 and AA6 on TcdA while not clashing with the toxin by itself. We suggest that, guided by the known binding epitopes and the relative positions of these four VHHs on TcdA and TcdB holotoxin, the designs of these multivalent VHHs could be further optimized to ensure simultaneous binding of AH3/AA6 on TcdA and 5D/E3 on TcdB, which should lead to even higher neutralizing potency.

The identification of these two neutralizing epitopes also provides new insight for developing fragment-based vaccines against TcdA. The large size of TcdA and TcdB poses a challenge in developing vaccines, as they induce large numbers of non-neutralizing antibodies. Prior studies have demonstrated that non-neutralizing antibodies could enhance cytotoxicity and inflammation *via* antibody-mediated entry into cells (26, 62). In fact, various toxin fragments have previously been explored as vaccine candidates (63–67). We envision that these two newly identified neutralizing epitopes in TcdA are good candidates for the development of fragment vaccines, which will focus the immune responses toward these functionally critical epitopes in TcdA, eliciting antibodies to block TcdA translocation and thereby shielding host cells from toxin attack.

## Data availability statement

The coordinates and structure factors for the TcdA GTD-AH3 and TcdA^1073-1464^-AA6 complexes have been deposited in the Protein Data Bank under access codes 7UBY and 7UBX, respectively.

## Author contributions

Conceptualization: BC and RJ; investigation: BC and KP; supervision and funding acquisition: RJ; writing: BC and RJ. All authors contributed to the article and approved the submitted version.

## Funding

This work was partly supported by National Institute of Allergy and Infectious Diseases (NIAID) grants R01AI139087, R01AI158503, and R21AI156092 to RJ. NE-CAT at the Advanced Photon Source (APS) is supported by a grant from the National Institute of General Medical Sciences (P30 GM124165). Use of the APS, an Office of Science User Facility operated for the U.S. Department of Energy (DOE) Office of Science by Argonne National Laboratory, was supported by the U.S. DOE under Contract No. DE-AC02-06CH11357.

## Acknowledgments

We thank Dr. Charles B. Shoemaker at Tufts University for kindly sharing with us the genes encoding VHH AH3 and AA6.

## Conflict of interest

The authors declare that the research was conducted in the absence of any commercial or financial relationships that could be construed as a potential conflict of interest.

## Publisher’s note

All claims expressed in this article are solely those of the authors and do not necessarily represent those of their affiliated organizations, or those of the publisher, the editors and the reviewers. Any product that may be evaluated in this article, or claim that may be made by its manufacturer, is not guaranteed or endorsed by the publisher.
