# Supplementary material for: Neutralizing epitopes on Clostridioides difficile toxin A revealed by the structures of two camelid VHH antibodies
Source: Front Immunol. 2022 Nov 16;13:978858. doi: 10.3389/fimmu.2022.978858 (PMC9709291; doi:10.3389/fimmu.2022.978858)
Supplement: Supplementary file 1 [file DataSheet_1.pdf]

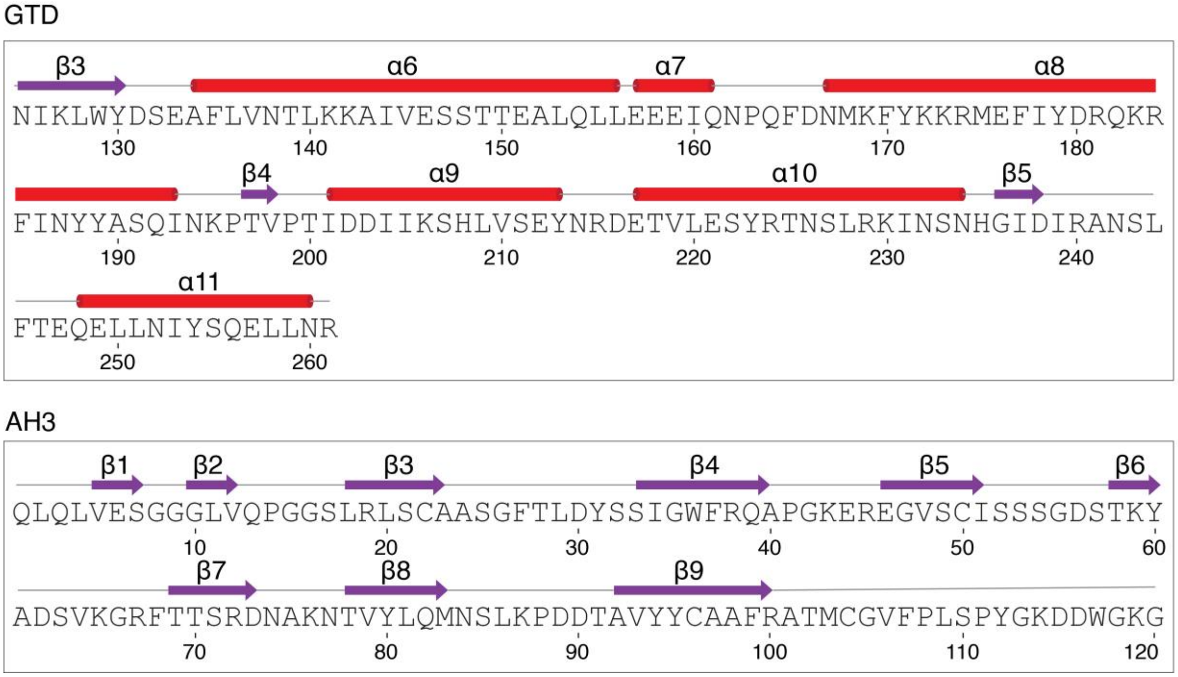

Supplemental Figure 1 | Schematic representation of the secondary structures focusing on the TcdA GTD-AH3 interface.

α-helix and β-sheet are shown as red cylinder and purple arrow, respectively.

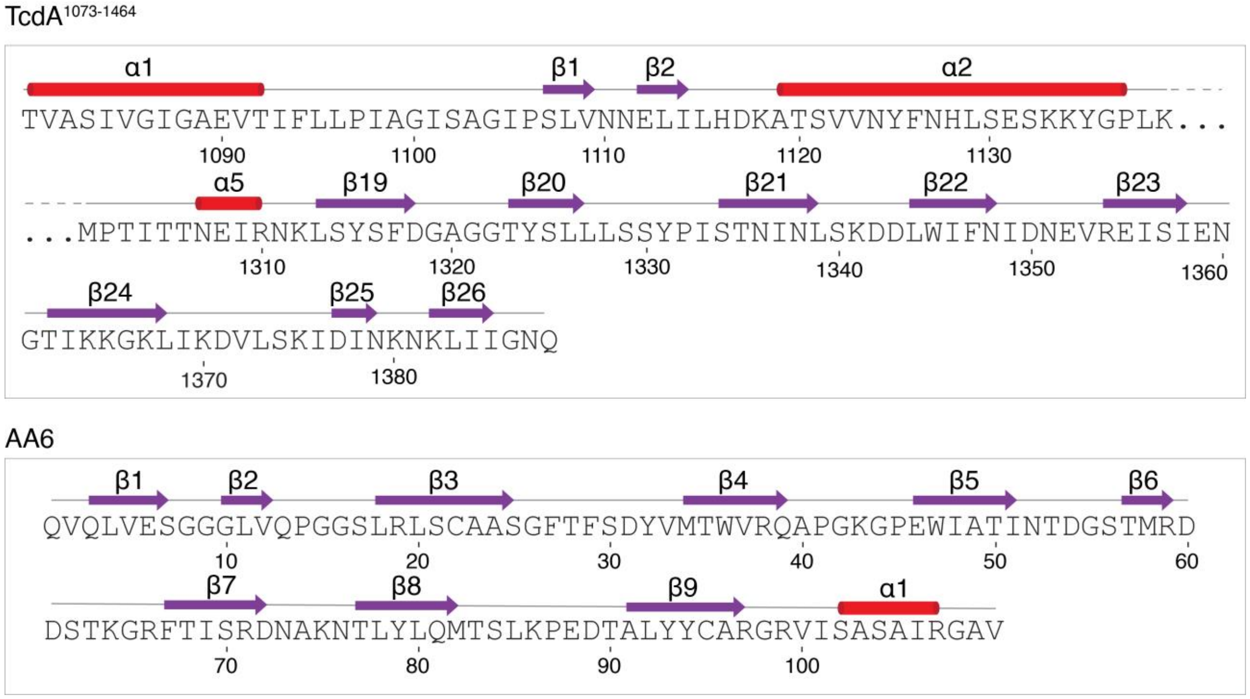

Supplemental Figure 2 | Schematic representation of the secondary structures focusing on the TcdA$^{1073\text{-}1464}$-AA6 interface.

α-helix and β-sheet are shown as red cylinder and purple arrow, respectively.

Table S1 Data collection, phasing, and refinement statistics.

**Data Collection**	**GTD-AH3**	**TcdA$^{1073-1464}$-AA6**
Space Group	P 1 21 1	P 1 21 1
Cell Dimensions		
a, b, c (Å)	80.12, 131.64, 83.80	54.02, 86.00, 109.34
α, β, γ (°)	90.00, 110.02, 90.00	90.00, 91.45, 90.00
Wavelength (Å)	0.9792	0.9792
Resolution (Å)	67.57 - 2.10 (2.17 - 2.10)	54.65 - 1.81 (1.88 - 1.81)*
R_{merge} (%)	0.068 (0.388)	0.059 (0.739)
R_{pim}	0.043 (0.253)	0.037 (0.480)
Wilson B-factor (Å^2)	33.22	31.12
Mean I/σ(I)	12.52 (2.52)	11.85 (1.39)
$CC_{1/2}$	0.997 (0.903)	0.998 (0.702)
Completeness (%)	99.28 (97.16)	98.00 (92.09)
Redundancy	3.5 (3.2)	3.4 (3.2)
**Refinement**		
Resolution (Å)	67.57 - 2.10 (2.17 - 2.10)	54.65 - 1.81 (1.88 - 1.81)
No. of reflections	94397 (9177)	88827 (8321)
R_{work}/R_{free}	21.8/26.1	19.5/23.7
No. of atoms	10704	8521
Protein	10231	7856
Ligand/ion	132	0
Water	341	665
B-factor (Å^2)	39.90	38.89
Protein	40.08	38.61
Ligand/ion	38.17	0
Water	35.00	42.24
Ramachandran plot		
Favored (%)	97.69	98.11
Allowed (%)	2.31	1.79
Outliers (%)	0.00	0.10
R.m.s. deviations		
Bond lengths (Å)	0.009	0.008
Bond angles (°)	0.960	1.010

* Values in parentheses are for the highest-resolution shell.

Table S2 Protein-protein interactions between AH3 and TcdA GTD.

AH3	TcdA GTD	Interaction Type
Q3/L4/A97	P196	vdW
F27	P196<br>T197	vdW
S32	T197	vdW
E44	Q248	vdW
K59	A241	vdW
A98	P196	HB (mc-mc), 3.5 Å
	T197	vdW
	V198	HB (mc-mc), 2.7 Å
F99	V198	vdW
R100	T197	vdW
	V198	HB (mc-mc), 3.0 Å
	P199	vdW
	T200	vdW
	D203	HB (sc-sc), 2.9 Å<br>SB, 3.8 Å
A101	E133<br>T200	vdW
G105	E133<br>R240	vdW
V106	R240<br>L251	vdW

AH3	TcdA GTD	Interaction Type
L109	F245<br>T246<br>E247<br>Q248<br>L251	vdW
S110	Q248	HB (mc-sc), 2.8 Å
	L251	vdW
P111	I193<br>V198	vdW
Y112	Y189	HB (mc-sc), 3.8 Å
	I193	vdW
	N252	vdW
	S255	vdW
	Q256	vdW
	L259	vdW
	N260	HB (sc-sc), 2.8 Å
G113	I193	vdW
	N194	HB (mc-sc), 3.3 Å
K114	I193	HB (sc-mc), 3.4 Å
	N194	vdW
	K195	HB (sc-mc), 3.1 Å
	V198	vdW
D115/D116	N194	HB (mc-sc), 2.9/2.8 Å

"HB", "SB", "vdW" stand for hydrogen bond, salt bridge, and van der Waals interaction, respectively. "mc" indicates the main-chain mediated contacts, and all the other contacts are mediated by side-chain atoms.

Table S3 Mapping the interactions between AA6 and TcdA fragments.

TcdA fragments	Biochemical behavior *	Pull down by His-AA6
1-1832	Monodispersed	Yes
843-2481	Monodispersed	Yes
1043-1802	Monodispersed	Yes
1043-1475	Monodispersed	Yes
1073-1802	Monodispersed	Yes
1073-1475	Monodispersed	Yes
1073-1464	Monodispersed	Yes

* The biochemical behavior was judged based on Superdex-200 size-exclusion chromatography.

Table S4 Protein-protein interactions between AA6 and TcdA$^{1073\text{-}1464}$.

AA6	TcdA$^{1073\text{-}1464}$	Interaction Type
D31	K1370	vdW
Y32	K1370	vdW
	D1371	HB (sc-sc), 3.1 Å
V33	E1355	vdW
	I1369	
W47	N1307	HB (mc-sc), 2.9 Å
	R1310	vdW
	Y1331	vdW
N52	E1355	vdW
	I1356	HB (sc-mc), 3.0 Å
	S1357	vdW
T53	E1355	HB (mc/sc-sc), 2.7/3.9 Å
D54	E1355	vdW
S56	S1357	vdW
T57	I1356	vdW
	S1357	
	I1358	
M58	S1330	vdW
	Y1331	
	I1356	
R59	T1305	vdW
	Y1331	HB (mc-sc), 3.6 Å
D60	T1305	vdW
	T1306	vdW
	N1307	HB (sc-mc), 3.0 Å
D61	I1105	vdW
	P1106	vdW
	T1305	HB (sc-sc, mc-mc), 2.5/2.9 Å
	T1306	vdW
S62	L1108	vdW
	T1306	
R66	L1108	vdW
E88	N1110	vdW
	N1111	
R99	S1330	vdW
	N1351	HB (sc-mc), 3.1 Å
	E1352	HB (sc-mc), 3.1 Å
	V1353	vdW
	R1354	HB (sc-mc), 2.8 Å
	E1355	vdW
	I1356	vdW
	N1387	HB (mc-sc), 2.9 Å
V100	N1387	vdW
I101	R1310	vdW
	S1330	vdW
	P1332	vdW
	N1387	HB (mc-sc), 2.8 Å
S102	G1386	vdW
	N1387	HB (mc-sc), 3.4 Å
S104	I1384	vdW
A105	G1386	vdW
	N1387	

“HB”, “SB”, “vdW” stand for hydrogen bond, salt bridge, and van der Waals interaction, respectively. “mc” indicates the main-chain mediated contacts, and all the other contacts are mediated by side-chain atoms.
